# Socioeconomic status and hospitalization in the very old: a retrospective study

**DOI:** 10.1186/1471-2458-7-227

**Published:** 2007-08-31

**Authors:** Raffaele Antonelli-Incalzi, Carla Ancona, Francesco Forastiere, Valeria Belleudi, Andrea Corsonello, Carlo A Perucci

**Affiliations:** 1 Chair of Geriatric Medicine, University Campus Bio-Medico, Rome, Italy; 2 San Raffaele Foundation, Cittadella della Carità, Taranto, Italy; 3 Department of Epidemiology, Local Health Authority RME, Rome, Italy; 4 Italian National Research Center on Aging (INRCA), Cosenza, Italy

## Abstract

**Background:**

Socioeconomic status could affect the demand for hospital care. The aim of the present study was to assess the role of age, socioeconomic status and comorbidity on acute hospital admissions among elderly.

**Methods:**

We retrospectively examined the discharge abstracts data of acute care hospital admissions of residents in Rome aged 75 or more years in the period 1997–2000. We used the Hospital Information System of Rome, the Tax Register, and the Population Register of Rome for socio-economic data. The rate of hospitalization, modified Charlson's index of comorbidity, and level of income in the census tract of residence were obtained. Rate ratios and 95% confidence limits were computed to assess the relationship between income deciles and rate of hospitalization. Cross-tabulation was used to explore the distribution of the index of comorbidity by deciles of income. Analyses were repeated for patients grouped according to selected diseases.

**Results:**

Age was associated with a marginal increase in the rate of hospitalization. However, the hospitalization rate was inversely related to income in both sexes. Higher income was associated with lower comorbidity. The same associations were observed in patients admitted with a principal diagnosis of chronic condition (diabetes mellitus, heart failure, chron obstructive pulmonary disease) or stroke, but not hip fracture.

**Conclusion:**

Lower social status and associated comorbidity, more than age per se, are associated with a higher rate of hospitalization in very old patients.

## Background

As the population ages, patients over 64 account for a continuously growing proportion of acute hospital care [1]. In Lazio, the region surroundings Rome, people over 64 accounted for 44.6% of hospital stays in 1996 and 49.3% in 2002 [2]. Concern has been raised about the economic implications of the "geriatric epidemic", and the older people are considered to be responsible for an extraordinary consumption of health care resources [3]. However, a recent analysis limited to patients who died in hospital in the UK showed that age was not associated with a greater number of days spent in the hospital in the year preceding death [4]. Furthermore, a German study demonstrated that the average number of hospital days for the last year of life was stable in people who die between 50 and 90 years of age, but significantly higher for persons who die at younger ages [5]. Thus, the growing number of older people in the general population, and not increased use of health care resource by individual old patients, seems to be responsible for the impact ageing has on health care expenditures. However, this conclusion stems from studies that used only age as a potential determinant of hospital bed utilization.

Besides being a relevant correlate of self-rated health status, functional status, morbidity and mortality [6-9], social inequalities could also affect the demand for acute hospital care. Rates of any hospitalization is higher in subjects with low socioeconomic status (SES) than among those living in high SES neighborhood aged 25 to 74 years [10]. Furthermore, the risk of hospitalization for heart failure among those aged 45 to 64 years is 39% greater in the most versus least socially and economically deprived subjects in a population, irrespective of baseline cardio-respiratory status and cardiovascular risk factors [11]. However, such a strict relationship between low SES and hospitalization is less certain in people aged 75 and over. Because comorbidity dramatically increases with age [12], comorbidity and not SES could represent the major determinant of the need of hospital care in the very old.

We planned the present study to evaluate whether socioeconomic status, as measured by proxy variable such as area-based income, affects acute hospitalization rates also in very old people.

## Methods

### Data source and selection criteria

We examined the discharge abstracts data of acute care hospital admissions of residents in Rome aged 75+ years in the period 1997–2000. Discharge abstract data are routinely collected by the regional Hospital Information Systems (HIS) and include: patient demographic data, admission and discharge dates, admission referral source, discharge status, up to six discharge diagnoses (ICD-9-CM), up to six hospital procedures (ICD-9-CM), regional code of the facility, up to four in-hospital transfers, and date of in-hospital transfer. The information system covers all hospitals in the region and includes also hospitalizations of residents occurred outside the region. The study protocol was approved by the Ethical Committee of the Local Health Authority RME, Rome, Italy.

### Income data

As a surrogate of individual socioeconomic status, we considered the income level of the population living in the census tract (CT) of residence. A median familiar equivalent income index has been derived for each of the 5736 census tracts (CT) of Rome (average population = 480 inhabitants) [13]. In synthesis, data relative to income earned in 1998 (tax returns of the year 1999) were extracted from the Italian Tax Register for all residents of Rome as of the 1^st ^of January 1998. A record linkage between the Tax Register and the Population Register of Rome connected family status information to income data for each subject, then the family equivalent income, weighted for the number of family members was calculated. Data were aggregated at the CT level, and the median value for each CT was calculated. Due to confidentiality of information, only details about income for each CT were available in our study database

In order to obtain categorical values for the income indicator, we calculated the deciles of the income distribution (1^st ^decile very underprivileged, 10^th ^decile very well off) on the basis of the whole adult population.

### Hospitalization rates

We computed age-standardised rates of hospitalization (per 1000 inhabitants) by gender and income decile for the three age groups ≥ 75 years, 75–84 years, and ≥ 85 years. The cut off of 75 years was chosen because it marks a dramatic increase in the prevalence of comorbidity and disability [14]. We used all persons residing in Rome at the 1^st ^of January, for each year under study, as the denominator population. All rates were directly standardised using the population of Italy for 1998 as reference. In addition to overall hospitalization, we analysed the following: diabetes (ICD-IX code 250), heart failure (ICD-IX code 428), stroke (ICD-IX codes 431, 432, 434, 436), Chronic Obstructive Pulmonary Disease (COPD) (ICD-IX codes 491, 492), and hip fracture (ICD-IX code 820). Three of these conditions (COPD, heart failure, diabetes mellitus) may be considered as ambulatory care sensitive conditions, i. e. high hospitalization rate for these chronic conditions suggests that community health care is inappropriate [15]. Instead, stroke and hip fracture are acute conditions requiring hospitalization irrespective of primary health care. It should be noted that we took into account only hospitalizations in acute care public or private hospitals and not in rehabilitation or day hospital.

To quantify the burden of comorbidity, i. e. of diseases coexisting with the main disease, during the hospital admission, we computed for each hospitalized subject a modified version of the Charlson Index of comorbidity: individual diagnoses codified according to ICD-9-CM [16] were given a score proportional to the diagnosis-related risk of death [16,17]. For each patient the final score was obtained by summing the scores of individual diagnoses. For instance, a patient having COPD as the main disease and peripheral vascular disease (codes 443.9, 441–441.9, 785.4, V43.4) and diabetes with chronic complications (codes 250.4–250.6) as comorbid diseases had a Charlson index of 3 corresponding to the sum of 1, the score of peripheral vascular disease, and 2, the score of diabetes with chronic complications. A detailed list of diagnoses and corresponding scores is available in the reference [17].

We used Rate Ratios (RRs) to compare hospital admission rates among income deciles, using the first income decile (the lowest) as the reference group. Confidence intervals (CI) were calculated at the 95% level of significance by using the standard error of the age-adjusted rates. We used multiple linear regression analysis to evaluate the association between the log transformation of duration of hospital stay with income deciles among men and women. Age was considered in the regression models. Statistical analysis was performed using STATA 8 statistical software package.

## Results

Age-standardised rates of hospitalization by income decile, separately for males and females, are reported in Table 1. There was only a marginal increase in the hospitalization rate from 75–84 to 85+ year old people in males and in females. The difference was lower when fatal hospital admissions (admissions which ended with a fatal outcome) were excluded from the analysis (hospitalization rates per 1000 (CI 95%): males 75–84 years = 386 (384–389), 85+years = 384 (379–389); females 75–84 years = 285 (284–287), 85+ years = 309 (306–312)). On the other hand, the rate of hospitalization dramatically increased for decreasing income in both sexes and age groups. The increase in hospitalisation rate from 75–84 to over 85 years was greater in the lowest than in the highest SES group. For instance, such an increase for women in the 1^st ^decile of SES (481-397 = 84) was greater than for women in the 10^th ^decile of SES (283-246 = 37).

**Table 1:** Hospitalization rates (per 1000) by age group, income decile, and gender.

	Age groups											
	75+				75–85				85+			
			
**Males**												
Income decile	Pop.	Rates	95% CI		Pop.	rates	95% CI		Pop.	rates	95% CI	
1	20847	557	547	568	16631	552	541	564	4216	572	549	595
2	22508	511	501	520	18072	491	481	501	4436	567	545	590
3	25700	487	478	495	20439	469	460	478	5261	536	517	557
4	27910	459	451	468	22235	451	443	460	5675	482	464	501
5	32382	439	431	446	25483	424	416	432	6899	480	464	496
6	31543	433	425	440	24951	416	408	424	6592	479	462	496
7	30348	414	407	422	23605	409	400	417	6743	431	415	447
8	38776	393	385	400	21899	387	379	395	6877	408	393	423
9	30430	371	364	378	23104	367	359	374	7326	383	369	398
10	34515	343	337	350	25759	337	330	344	8756	362	350	375
all	284959	432	430	434	222178	454	451	457	62781	456	451	461
		*RR 1vs10*	*95%CI*			*RR 1vs10*	*95%CI*			*RR 1vs10*	*95%CI*	
		1.62	1.58	1.67		1.64	1.59	1.69		1.62	1.56	1.68
	
**Females**												
Income decile	Pop.	Rates	95% CI		Pop.	rates	95% CI		Pop.	rates	95% CI	

1	41768	419	413	425	30622	397	390	404	11146	481	469	494
2	42850	377	371	383	31256	359	352	365	11594	429	418	441
3	46567	351	346	356	34018	330	324	336	12549	410	399	421
4	51071	352	347	357	37380	332	326	337	13691	410	400	421
5	59147	325	321	330	42311	308	303	313	16836	374	365	384
6	58349	316	311	321	41557	302	297	307	16792	356	347	365
7	57165	305	301	310	40536	290	285	295	16629	349	340	358
8	53453	295	290	299	37270	282	277	288	16183	330	322	339
9	57785	276	271	280	39582	263	258	268	18203	312	304	320
10	60991	256	252	260	41034	246	242	251	19957	283	276	290
all	529146	322	320	323	375566	334	332	336	153580	364	361	367
		*RR 1vs10*	*95%CI*			*RR 1vs10*	*95%CI*			*RR 1vs10*	*95%CI*	
		1.64	1.60	1.67		1.61	1.57	1.65		1.62	1.56	1.68

**Figure 1:**
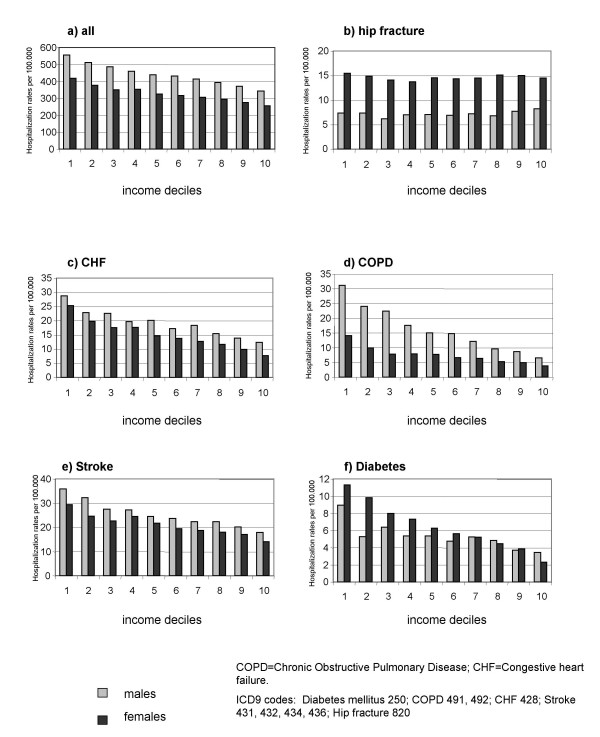
Rate of hospitalization for selected conditions in a population aged over 74 years.

The same trend in age-standardised rates of hospitalization by income decile was observed in patients with diabetes mellitus (1^st ^vs 10^th ^income decile, males RR = 2.59, 95% CI = 2.05–3.27, females RR = 4.92, 95% CI = 4.07–5.94), heart failure (males RR = 2.32, 95% CI = 2.04–2.63, females RR = 3.28, 95% CI = 2.95–3.65), COPD (males RR = 4.31, 95% CI = 3.74–4.97, females RR = 3.28, 95% CI = 2.85–3.77) or stroke (males RR = 1.93, 95% CI = 1.74–2.13, females RR = 2.07, 95% CI = 1.91–2.25), but not in those with hip fracture (males RR = 0.89, 95% CI = 0.73–1.10, females RR = 1.07, 95% CI = 0.97–1.18) (Figure 1).

In both men and women, there was a decreasing cumulative number of days spent in the hospital with the increase in area-based income. The median length of stay was 12 (SD 12.8) and 12.9 (SD 14.4) days, for men and women respectively, among those in the lowest income decile, and 9.7 (SD 11.5) and 11.3 (SD 13.2) days among those in the highest income decile. When we adjusted for age in the multivariate linear regression analysis, the strong statistically significant inverse relationship remained (p < 0.001).

When comorbidity was examined among hospitalized individuals, higher income was associated with low comorbidity in both genders (Table 2 and 3).

**Table 2:** Distribution of Charlson index (%) by principal diagnosis and income decile in males.

	all				Diabetes mellitus				COPD*				CHF**				Stroke				Hip fracture			
		0	1	1+		0	1	1+		0	1	1+		0	1	1+		0	1	1+		0	1	1+

i.d. °	n	%	%	%	n	%	%	%	n	%	%	%	n	%	%	%	n	%	%	%	n	%	%	%
1	11553	36.5	30.3	33.2	159	43.4	37.1	19.5	649	66.6	23.1	10.3	573	38.6	37.3	24.1	750	55.3	28.3	16.4	137	73.7	16.8	9.5
2	11354	37.5	28.1	34.4	104	56.7	25.0	18.3	541	70.6	19.4	10.0	490	35.5	38.2	26.3	728	55.9	27.6	16.5	147	70.7	19.7	9.5
3	12356	38.7	29.3	32.0	141	46.1	36.9	17.0	575	70.8	20.9	8.4	566	38.0	37.3	24.7	709	59.2	27.9	12.8	145	71.0	16.6	12.4
4	12744	39.9	28.3	31.9	120	48.3	26.7	25.0	489	67.1	23.9	9.0	535	40.2	35.3	24.5	760	55.9	27.1	17.0	180	71.1	18.3	10.6
5	14085	39.7	27.7	32.6	157	47.8	31.2	21.0	488	72.8	19.1	8.2	624	43.9	31.7	24.4	795	56.7	29.4	13.8	214	72.0	19.6	8.4
6	13492	40.7	26.8	32.5	132	48.5	26.5	25.0	467	68.5	20.1	11.4	522	41.8	33.9	24.3	750	57.5	25.6	16.9	202	81.7	13.4	5.0
7	12530	41.4	26.1	32.5	146	44.5	39.0	16.4	369	66.1	23.9	10.0	537	40.2	36.3	23.5	677	58.9	23.8	17.3	202	80.7	11.9	7.4
8	11265	43.2	25.6	31.2	124	52.4	25.0	22.6	275	66.2	25.8	8.0	439	39.2	39.9	21.0	645	62.8	23.4	13.8	188	77.1	14.9	8.0
9	11262	43.9	25.2	30.9	104	44.2	35.6	20.2	263	71.5	20.2	8.4	414	46.1	30.0	23.9	616	62.8	22.4	14.8	227	76.7	16.7	6.6
10	11830	48.4	23.0	28.5	90	55.6	25.6	18.9	224	70.1	18.8	11.2	424	49.3	30.2	20.5	621	63.5	22.2	14.3	278	80.2	14.0	5.8

* Chronic Obstructive Pulmonary Disease; **Congestive Heart Failure. ICD IX codes: Diabetes mellitus 250; COPD491,492; CHF 428; Stroke 431, 432, 434, 436; Hip fracture 820. ° income decile

**Table 3:** Distribution of Charlson index (%) by principal diagnosis and income decile in females.

	all				Diabetes mellitus				COPD*				CHF**				Stroke				Hip fracture			
		0	1	1+		0	1	1+		0	1	1+		0	1	1+		0	1	1+		0	1	1+

i.d. °	n	%	%	%	n	%	%	%	n	%	%	%	n	%	%	%	n	%	%	%	n	%	%	%
1	17488	43.6	31.6	24.8	421	56.8	33.5	9.7	590	68.6	24.1	7.3	1057	50.1	33.5	16.4	1230	62.5	24.2	13.3	648	80.1	15.7	4.2
2	16161	45.9	29.4	24.7	378	57.7	29.9	12.4	426	65.7	27.5	6.8	853	50.6	35.1	14.3	1057	60.6	26.4	13.1	640	81.4	13.9	4.7
3	16334	47.2	28.9	23.9	324	56.8	29.9	13.3	363	70.5	24.2	5.2	815	51.5	36.0	12.5	1057	63.3	23.0	13.7	660	79.2	16.5	4.2
4	17989	47.2	29.6	23.2	337	58.8	28.8	12.5	405	72.1	23.5	4.4	903	54.3	31.3	14.4	1258	65.6	22.6	11.8	704	82.5	12.5	5.0
5	19331	49.0	27.4	23.6	323	55.1	32.5	12.4	455	68.6	24.6	6.8	880	51.0	32.3	16.7	1288	60.6	27.2	12.2	882	83.7	13.4	2.9
6	18507	49.7	26.6	23.7	291	52.6	32.3	15.1	385	64.7	26.8	8.6	819	54.5	30.5	15.0	1140	62.5	25.7	11.8	855	82.9	13.9	3.2
7	17547	50.1	26.6	23.2	265	52.1	32.5	15.5	364	70.9	21.2	8.0	741	53.4	31.2	15.4	1072	66.0	21.4	12.6	852	84.4	12.0	3.6
8	15862	51.9	25.2	22.9	212	58.0	29.2	12.7	282	71.6	20.9	7.5	645	48.2	35.3	16.4	968	65.0	20.5	14.6	839	86.4	10.0	3.6
9	16084	53.5	25.6	21.0	202	56.9	31.2	11.9	286	74.5	21.0	4.6	604	58.9	28.3	12.7	988	68.7	19.5	11.7	919	86.3	10.8	2.9
10	15759	55.8	23.8	20.4	121	61.2	26.4	12.4	233	79.0	16.7	4.3	499	57.9	32.9	9.2	862	71.4	19.0	9.6	937	88.8	9.0	2.2

* Chronic Obstructive Pulmonary Disease; **Congestive Heart Failure. ICD IX codes: Diabetes mellitus 250; COPD491,492; CHF 428; Stroke 431, 432, 434, 436; Hip fracture 820. ° income decile

## Discussion

Our data show that lower social status, more than age, is correlated with the rate of hospitalization in a population older than 74 years. A longer hospital stay was also detected in the lowest socioeconomic group when compared with those in the upper income category. Comorbidity also was greater in low income patients admitted to the hospital. Thus, socioeconomic inequalities are relevant to explain differences in health care use also in a very old population.

In keeping with our findings, a study conducted in UK showed that an elderly population tenants had a higher institutionalisation rate than owner-occupiers, who represent a higher income population [18]. Furthermore, lower socioeconomic status has been reported to be associated with excess hospitalization of diabetic patients for hypoglycaemia or hyperglycaemia, and this association was independent of age and comorbidity, but it was stronger in middle aged than in elderly diabetics [19]. Analogously, lower income Congestive Heart Failure patients are known to experience a greater rate of hospitalization [20].

The inverse association between income level and hospitalization rate may reflect two concurrent phenomena: higher incidence and prevalence of diseases among people in less advantaged conditions, and inadequate community care, especially secondary care, among poor people resulting in higher demand for hospitalization. The former phenomenon is testified by lower comorbidity and healthier life style characterizing high income subjects in developed countries [21]. Compliance to prescribed treatment is greater in more educated and affluent patients, and this might further reduce both the risk of stroke and the need for hospital care [22]. Furthermore, better income is associated with more efforts to fight modifiable risk factors such as obesity and smoking as well as with lesser exposition to stressors [23-25], which are an important risk factor for several diseases. Finally, education per se has been suggested to have a protective role against cognitive decline [26]. Besides being negatively associated with selected risk factors for chronic diseases, higher income also is a marker of better access to the health care facilities [27]. Interestingly, low income and poor education are associated with greater use of primary care services and lesser of secondary care services, i. e. with a gap between primary and hospital care [28,29]. This conclusion is likely true even for an universal and free health system such as the Italian Health National System, because the proper use of health care resources largely depends upon patient factors, which in turn partly reflect education and social status, rather than on supply factors [30]. Furthermore, the access to freely available services may be hampered by logistic barriers (such as long waiting lists, poor availability of selected services in some health care district, or mobility problems) which affluent people can more easily overcome [31].

At variance from chronic diseases and stroke, the rate of hospitalization for hip fracture was not associated with income, as if the risk of fall were independent from socio-economic status. This finding is unlikely to suffer from "collection bias" because hip fracture requires hospital care and, thus, the recorded figures cannot be biased by alternative home care. Thus, it is a true finding which contrasts with most of previous observations showing that different measures of income are inversely correlated with the incidence of hip fracture [32-34]. However, admissions for both hip fracture and appendicitis, two non-ambulatory care-sensitive conditions, have been reported to be independent from socioeconomic status in a population based cohort study of diabetic patients study [19]. Also an ecological English study found no association between income and hip fracture, but an inverse one between income and risk of fall [35]. Differences in studied population, income quantification and study design might account for the observed discrepancy. Furthermore, elderly Italians, even the least affluent, seem to be characterized by better dietary patterns and nutritional status than elderly living in other European countries [36], and this might smooth the income-related difference in the risk of traumatic hip fracture [34,37]. Finally, the fact that hospitalization for stroke, an acute event like hip fracture, was inversely related to income does not conflict with evidence pertaining to hip fracture because the two conditions have different profiles of risk. Overall, the awareness of cardiovascular and metabolic risk factors for stroke is more widespread than that of risk factors for osteoporosis and hip fracture: for example, hypertension was regularly treated in 75% of hypertensive patients, while only 56.3% of patients with osteoporosis received active treatment in former studies [38,39]. The well proved association between higher socio-economic status and better control of risk factors for stroke might translate in more effective prevention of stroke in affluent people [40].

Some limitations of this study should be cited. First, the Charlson's index quantifies comorbidity and was available only among hospitalized subjects. Therefore, it was not possible to evaluate the effect of income while "adjusting" for comorbidity level. Furthermore, Charlson's index only to some extent assesses the severity of illness, which might be relevant to explain the observed pattern of hospitalization. Indeed, computing an index of disease severity would require a detailed clinical information which is not available on administrative databases. Second, we had no information on the type or cost of care as a function of age. However, there is a consistent evidence that aging is associated with under treatment of many conditions [41-43]. Accordingly, the rate of hospitalization and the cumulative number of days spent in the hospital are expected to provide a good measure of the cost of hospital care for the elderly, but they might overestimate the costs for the very old. Third, it cannot be excluded that socio-economic inequalities also indirectly affect the access to hospital care, e. g. subjects living alone might experience some delay in care for acute conditions and, thus, would have greater chances of dying at home without living any track in the Hospital Information System. Unfortunately, we had no information on the living arrangement and, then, could not take into consideration this potential source of bias. Eventually, we rated SES on the basis of an area-based measure because individual data were not available due to privacy issues. This might lead to some misclassification of SES. However, since the census blocks in Rome are rather small (few hundreds of inhabitants), the misclassification, if any, is likely to be minor. Furthermore, area-based socio-economic measures were proved to detect socio-economic disparities in mortality among subjects over 65 [44].

## Conclusion

Our findings add to previous observations by showing that income can conveniently target subjects at greater risk of hospitalization even in the very old population, while age per se cannot. Accordingly, measures of income might help targeting older people who could benefit the most from dedicated health care programs. Income data are easily available in administrative databases for the whole population and qualify as a cumulative index of health status or health risk, whereas medical databases are not so capillary in most countries and, thus, provide information on a minority of the population.

Efforts are needed to identify factors mediating the relationship between income and health status. Interventions contrasting individual mediators are highly desirable, but, in a broader perspective, attempts at removing social inequalities would be the main health care intervention. Such an intervention would decrease the need for hospital care, and this would translate in an important saving of resources. Thus, physicians, health care managers and political authorities should be aware that medical and social dimensions interact to determine health status and health care needs also in the very old. This underscores the need for a comprehensive view of the health needs and an integrated approach to them.

## Competing interests

The author(s) declare that they have no competing interests.

## Authors' contributions

RAI, CA, FF, VB, and CAP planned and conducted the study, performed the statistical analysis, and drafted the first version of the manuscript. AC contributed to the study design and to the final version of the manuscript.

## Pre-publication history

The pre-publication history for this paper can be accessed here:


